# Radiology examination as a diagnostic aid in presentations with wide differential diagnoses: Case report of new Hodgkin’s lymphoma on a background of poorly controlled HIV

**DOI:** 10.4102/sajr.v21i2.1239

**Published:** 2017-11-14

**Authors:** Rachel Hubbard, Jalpa Kotecha, Thomas Nash, Yu Jin Lee, Nasir Khan, Farhat Kazmi

**Affiliations:** 1 Radiology Department, Chelsea and Westminster Hospital, United Kingdom; 2 HIV Department, Chelsea and Westminster Hospital, United Kingdom; 3 Acute Assessment Unit, Chelsea and Westminster Hospital, United Kingdom

## Abstract

Hodgkin’s lymphoma and disseminated *Mycobacterium avium* complex (MAC) infection share similar clinical features; both may affect human immunodeficiency virus (HIV)-positive individuals. We discuss a patient with poorly controlled HIV-infection presenting with chest sepsis, dyspnoea and weight loss. Whilst the initial working diagnosis was that of MAC infection, pathology results had not met diagnostic criteria. Lymph node biopsy instead revealed classical Hodgkin’s lymphoma. We discuss the role of radiological examination in cases of diagnostic uncertainty.

## Introduction

Hodgkin’s lymphoma (HL) and disseminated *Mycobacterium avium* complex (MAC) infection are seen in patients who are human immunodeficiency virus (HIV)-positive. They often share similar clinical features such as fever, night sweats, lymphadenopathy, anaemia and weight loss. It may be difficult to distinguish these conditions clinically; mycobacterial cultures may only aid diagnosis after several weeks of incubation. Radiological imaging is therefore important to aid accurate, timely diagnosis and guide biopsy. We present a case illustrating a diagnostic dilemma in a patient presenting with poorly controlled HIV.

## Case report

A 55-year-old man presented to the Accident and Emergency Department with a 2-month history of dyspnoea (acutely worsening over 2 days preceding admission), cough productive of purulent sputum, fever and weight loss. He was known to be HIV-positive (genotype-2, diagnosed 1991) but had been non-compliant with antiretroviral medication (Truvada, Darunavir and Ritonavir) for 2 years. Additional background included a history of depression, subdural haematoma, recurrent bacterial chest infections and drug abuse (daily heroin smoker).

On examination, he was unkempt, cachectic and dehydrated. He was septic but maintaining normal oxygen saturations on room air. Cardiovascular and neurological examinations were unremarkable. He was tachypnoeic and crepitations were auscultated at the right lung base. His abdomen was non-tender with palpable hepatosplenomegaly. Right-sided anterior cervical and right-sided inguinal lymphadenopathy was palpated.

Blood tests revealed microcytic iron-deficient anaemia, lymphopaenia with white cell count 2.6 × 10^9^/L, C-reactive protein 198 mg/L, normal electrolytes and renal function, raised alkaline phosphatase level (1664 iU/L), hypoalbuminaemia (22 g/L) and deranged clotting. Lactate dehydrogenase was raised at 515 iU/L. Serology demonstrated serum Epstein–Barr Virus (EBV) DNA PCR of 26 900 and positive Hepatitis B core-antigen (e-Antigen negative). HIV-2 viral load was 720 and CD4 count was 28 cells/μL (3%).

**FIGURE 1:**
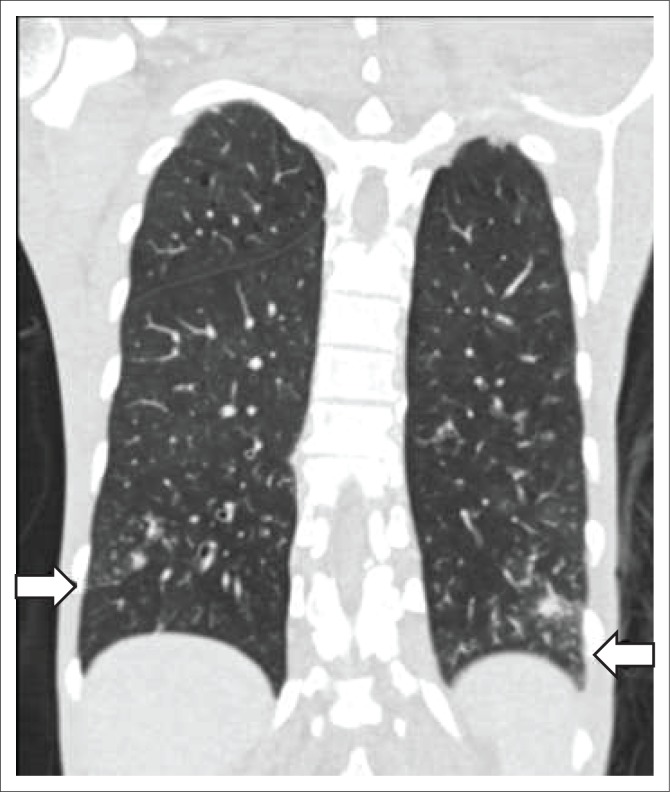
Coronal computerised tomography of the chest demonstrating bibasal lower lobe tree in bud appearance suspicious for TB.

**FIGURE 2:**
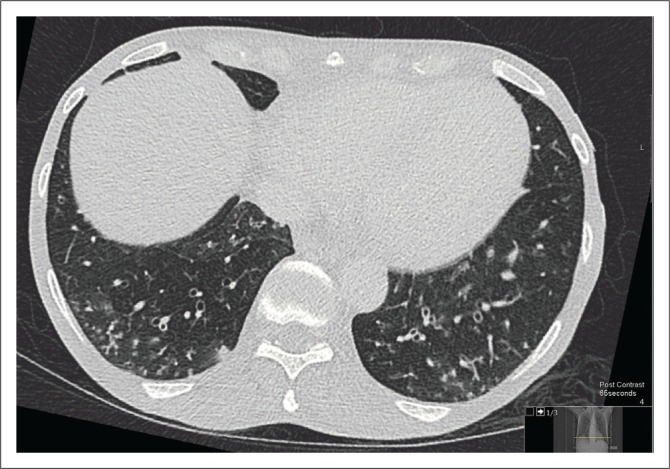
Axial computerised tomography of the chest demonstrating tree in bud appearances within the lung.

**FIGURE 3:**
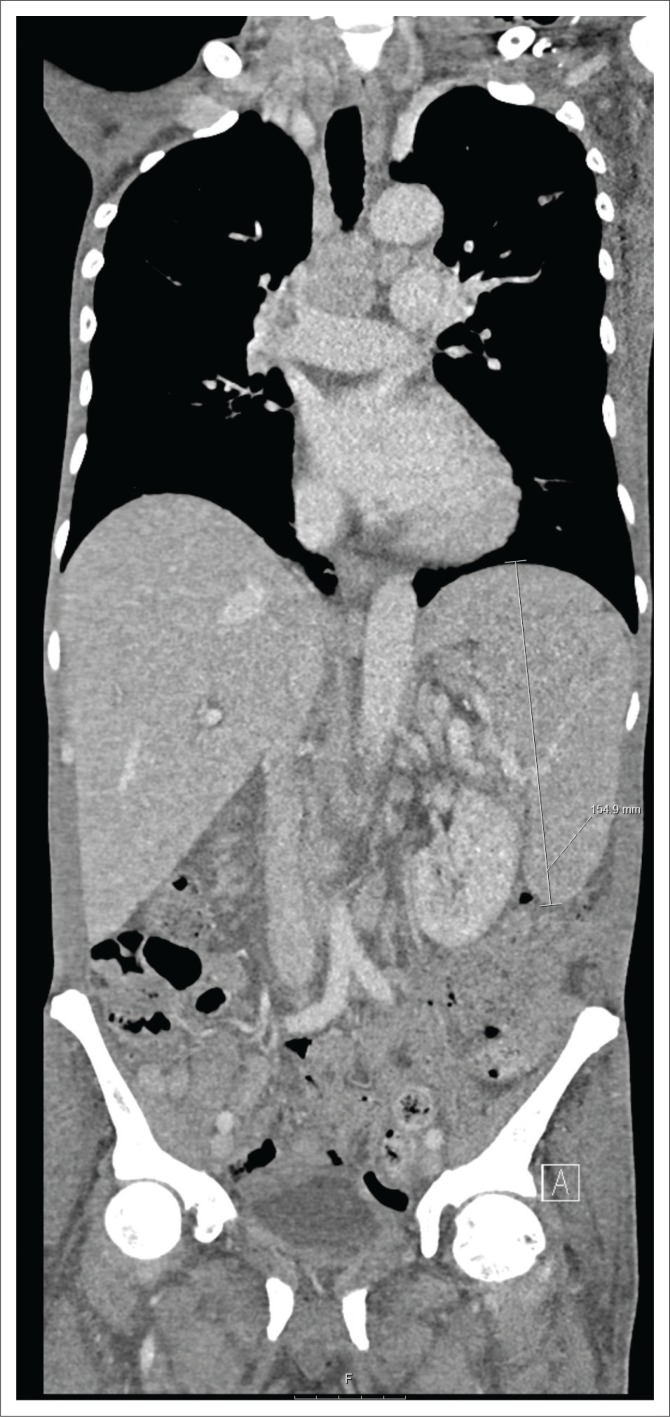
Coronal computerised tomography of the abdomen demonstrating hepatosplenomegaly.

**FIGURE 4:**
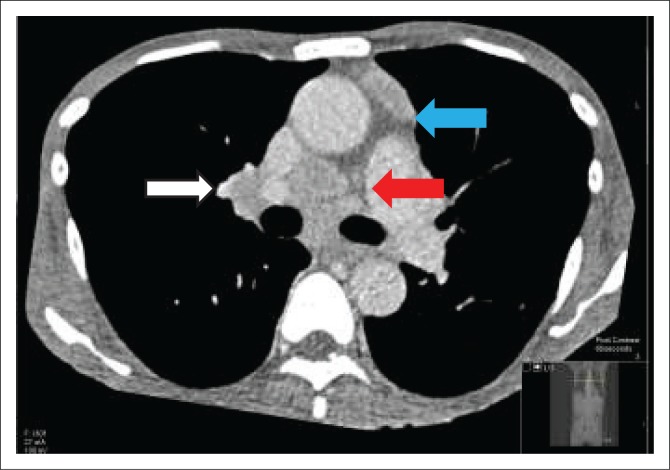
Axial computerised tomography of the chest demonstrating extensive mediastinal lymph nodes, the blue arrow points to the pre-vascular nodes, the white arrow points to the right hilar node and the red arrow points to the pre-carinal and subcarinal nodes.

**FIGURE 5:**
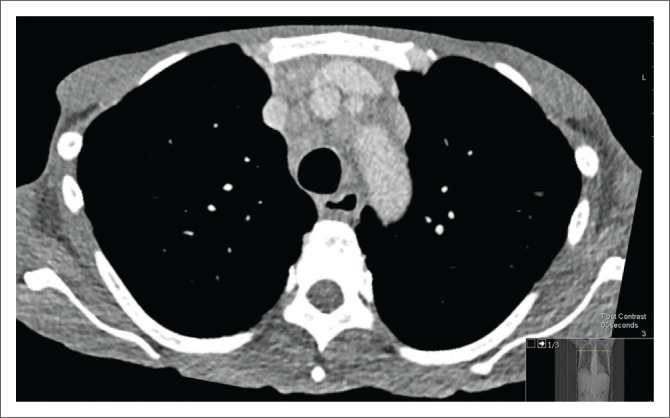
Anterior mediastinal lymph nodes.

A chest radiograph demonstrated right basal opacification. Computerised tomography of the chest abdomen and pelvis (CT-CAP) revealed patchy air space opacification at the left oblique fissure, left lower and right lower lobes, paraseptal and central lobular emphysema and bibasal atelectasis (Figures 1 and 2). Hepatosplenomegaly was confirmed and free fluid was evident throughout the abdomen and pelvis (Figure 3). Multiple enlarged lymph nodes (up to 1.8 cm diameter) were visualised throughout the thorax, abdomen and pelvis (Figures 4 and 5).

Sputum cultures identified *Pseudomonas aeruginosa*; acid alcohol fast bacilli (AAFB) smears were negative. Tests for respiratory viruses, atypical organisms and *Pneumocystis jirovecii* were negative. Urine and blood cultures were negative, with no growth on mycobacterial blood cultures up to that point. Optimised antiretroviral therapy was restarted alongside intravenous piperacillin and tazobactam.

On multi-disciplinary team meeting, the clinical case was deemed to be in keeping with a diagnosis of respiratory tuberculosis. Alternative differentials included atypical lower respiratory tract infection (including MAC infection) or lymphoma. The patient subsequently underwent supraclavicular lymph node biopsy, which revealed classical HL. A positron emission tomography CT (PET-CT) scan diagnosed ‘Stage 3B’ but suggested that some lymphadenopathy may have been secondary to concurrent HIV-related lymphadenopathy or infection. A staging CT head excluded cerebral metastases. He was consequently initiated on a regimen of Adriamycin, Bleomycin, Vinblastine and Dacarbazine (ABVD) chemotherapy.

Notably, one sputum culture subsequently grew *Mycobacterium intracellulare*; following this, another sputum culture grew *Mycobacterium mucogenicum*. Neither organism was grown on subsequent sputum samples. Neither of two mycobacterial blood cultures nor a lymph node mycobacterial culture showed any growth on final reporting. At least two sputum cultures positive for the same organism or one positive culture from a ‘sterile’ site (such as blood, lymph nodes or bone marrow) are required to diagnose atypical mycobacterial infection.^1,2^ The positive sputum cultures were therefore not considered significant.

## Discussion

The patient presented our multi-disciplinary team with a diagnostic challenge. Given his clinical presentation including low CD4 count, mycobacterial infection was felt to be the most likely diagnosis. However, radiological imaging highlighted significant, widespread lymphadenopathy throughout the thorax, abdomen and pelvis, which was later confirmed by tissue diagnosis to be HL.

Hodgkin’s lymphoma is more prevalent in HIV-positive individuals, and is often aggressive with B symptoms at presentation; it is associated with EBV-positivity.^3^ Interestingly, unlike other malignancies, its incidence has not decreased with the introduction of highly active antiretroviral therapy, although overall and progression-free survival has improved. CT-PET is superior to conventional CT for staging. However, biopsy is essential to confirm diagnosis.^4^

MAC (*M. avium* and *M. intracellulare*) accounts for up to 85% of infectious causes of abdominal lymphadenopathy in acquired immunodeficiency syndrome (AIDS).^5^ Whilst *M. avium* is common in disseminated MAC, *M. intracellulare* is a more important respiratory pathogen. MAC lung disease may present as either apical fibrocavitary disease or nodular and bronchiectatic changes (right middle lobe or lingula). The disease course is often indolent and it may be difficult to distinguish environmental contamination from clinically relevant infection. Consequently, bronchial lavage or biopsy is often required to aid diagnosis.^6^

*Mycobacterium mucogenicum* is typically seen in catheter-related and post-traumatic skin infections. It rarely causes respiratory tract infection, and positive sputum cultures are often because of environmental contamination.^7^

Widespread homogenous lymphadenopathy may be a feature of both MAC and HL; however, lymphadenopathy is often massive in HL (> 3 cm diameter).^5^ Hepatosplenomegaly is commoner in MAC than in HL.^5^ HL tends to be seen with a CD4 count around 240 cells/μL^4^; in contrast, disseminated MAC is typically found with CD4 < 50 cells/μL.^1^

HIV-2 tends to be less virulent with lower rates of progression to AIDS than in HIV-1; there are many differences in the behaviour of the virus and the consequent effects on the immune system that may explain this. The HIV-2 virus has a lower replication efficiency than HIV-1 and varies in its use of co-receptors. There are also differences in the levels and activity of the viral proteins Tat and Nef, which are thought to lead to a reduced rate of de novo T-cell infection and less immune activation (immune activation is strongly predictive of disease progression) in HIV-2 compared with HIV-1 infection.^8^ In HIV-2 infection, there are increased levels of CD4+CD25+ regulatory T-cells, an increased ability to replace infected CD4+ T-cells, and less T-cell apoptosis than in HIV-1 infection.^8^ Our patient, however, had poorly controlled, progressive HIV-2. This was likely because of his poor compliance with medication: he had stopped taking antiretrovirals on multiple occasions in the past and developed multiple resistances; his CD4 count had varied from 81 cells/μL – 370 cells/μL over the previous 12 years.

## Conclusion

HL and MAC present similarly in HIV-positive patients. Although the level of immunocompromise at which they present can differ, both represented feasible differential diagnoses in this case. In these situations, radiological assessment is essential in interpreting the clinical picture, guiding biopsy and, ultimately, achieving a confirmed diagnosis.

## Ethical consideration

No patient identifiable information has been presented. Written consent has been obtained from the patient prior to publication; a copy of the consent form has been provided.
